# General Prediction of Peptide-MHC Binding Modes Using Incremental Docking: A Proof of Concept

**DOI:** 10.1038/s41598-018-22173-4

**Published:** 2018-03-12

**Authors:** Dinler A. Antunes, Didier Devaurs, Mark Moll, Gregory Lizée, Lydia E. Kavraki

**Affiliations:** 1 Department of Computer Science, Rice University, Houston, TX 77005 USA; 2 Department of Melanoma Medical Oncology - Research, The University of Texas MD Anderson Cancer Center, Houston, TX 77054 USA

## Abstract

The class I major histocompatibility complex (MHC) is capable of binding peptides derived from intracellular proteins and displaying them at the cell surface. The recognition of these peptide-MHC (pMHC) complexes by T-cells is the cornerstone of cellular immunity, enabling the elimination of infected or tumoral cells. T-cell-based immunotherapies against cancer, which leverage this mechanism, can greatly benefit from structural analyses of pMHC complexes. Several attempts have been made to use molecular docking for such analyses, but pMHC structure remains too challenging for even state-of-the-art docking tools. To overcome these limitations, we describe the use of an incremental meta-docking approach for structural prediction of pMHC complexes. Previous methods applied in this context used specific constraints to reduce the complexity of this prediction problem, at the expense of generality. Our strategy makes no assumption and can potentially be used to predict binding modes for any pMHC complex. Our method has been tested in a re-docking experiment, reproducing the binding modes of 25 pMHC complexes whose crystal structures are available. This study is a proof of concept that incremental docking strategies can lead to general geometry prediction of pMHC complexes, with potential applications for immunotherapy against cancer or infectious diseases.

## Introduction

**Figure 1:**
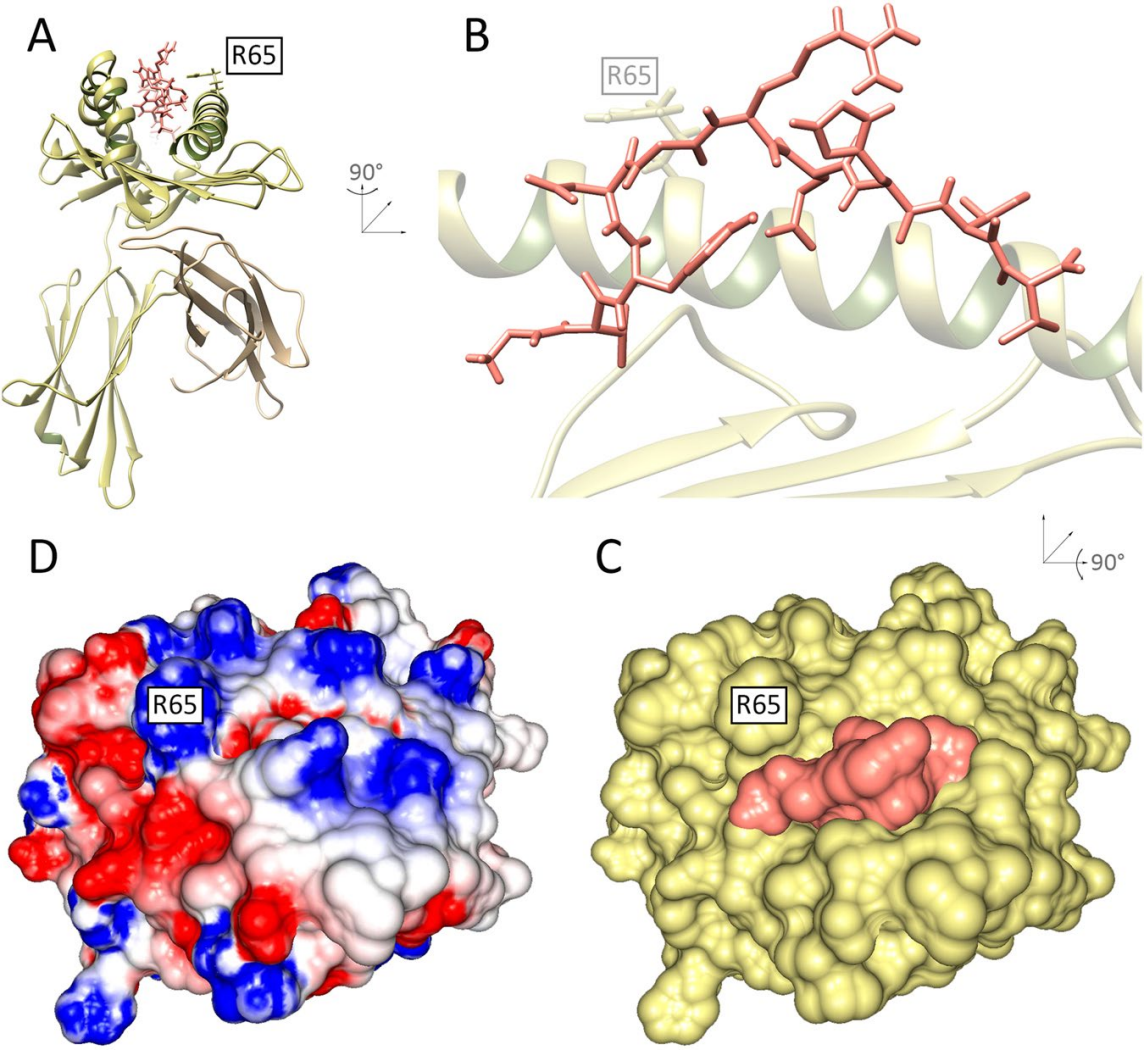
Structure of a pMHC complex. (**A**) Front view of the crystal structure 1I4F depicting a *cartoon* representation of the MHC (HLA-A*02:01), and a *sticks* representation of the bound peptide (derived from MAGEA4, in pink). The heavy chain of the MHC receptor (alpha), which contains the binding cleft, is depicted in yellow. The supporting light chain (*β*2-microglobulin) is depicted in brown. (**B**) Zoomed side view of a cross-section of the pMHC complex, highlighting the full length of the peptide in the MHC binding cleft. (**C**) Top view of the complex showing the *surface* of the MHC (yellow) and the exposed surface of the bound peptide (pink). (**D**) Combined surface of the pMHC complex, depicting the electrostatic potential distribution over the surface (red, negative regions; blue, positive regions); it is referred to as the “TCR-interacting surface” of the pMHC complex. For reference, the MHC amino acid residue number 65 (arginine, R65) is labeled in all views.

The so-called cellular immune response^1^ is based on a specific recognition system that is present in virtually every nucleated cell in the organism. As part of regular intracellular protein synthesis, some proteins are marked for degradation and proteolytically cleaved into smaller fragments (called peptides) that are then displayed at the cell surface^2^. The key molecules in this process are specialized protein-receptors known as class I major histocompatibility complexes (MHCs)^1,2^, which bind and display these intracellular peptides. Thanks to this peptide-presenting pathway, T-cell lymphocytes that circulate throughout the body scanning cell surfaces can monitor the intracellular content in almost every tissue. This allows for immune recognition of diseased cells (e.g., infected or tumoral). These peptide-MHC (pMHC) complexes (Fig. 1) can then be recognized by direct interaction with T-cell receptors (TCRs), activating T-cell cytolysis and triggering the elimination of the diseased cell^3^. Note that class II MHC molecules have a distinct structure^2^, are limited in expression to specialized immune cells, and are involved in a different pathway; they will not be discussed here.

Since a given class I MHC molecule can only bind a subset of existing peptides^4^, and since viral proteins have high mutation rates (yielding ever-changing peptide pools), MHC diversity became essential for the survival of the host population^1^. In fact, the MHC region is the most variable segment of the entire human genome: there are more than 8,000 known protein variants (or allotypes) of class I MHCs in the human population^5^, with up to 6 different allotypes per individual^1^. Besides its importance for anti-viral immunity and vaccine development, the recognition of pMHC complexes is a key factor in autoimmunity, response to tissue transplantation, and immunity against tumors^6,7^. In recent years, analyses of tumor-derived peptides capable of binding to patient-specific MHCs have played an essential role in the development of personalized immunotherapies against cancer^7^. Although many other molecules are involved in intercellular interactions between T-cells and tumor cells, the structural and biochemical properties of a given pMHC complex represent the central recognition feature and the most important information provided to the T-cell^6,8^ (Fig. 1). In the context of immunotherapy, this information will define the chances that activated T-cells find and eliminate cancer cells throughout the body^9^; it will also determine the occurrence of potentially lethal off-target toxicities against healthy tissues (referred to as T-cell cross-reactivity)^9–11^.

Given the importance of pMHC structural information in driving the cellular immune response, and the limitations of experimental methods for structural analyses of proteins, the structural prediction of pMHC complexes has been a desired goal in bioinformatics for over a decade^12–17^. Computational methods such as molecular docking are the most promising tools for this task, given their efficiency and broad use for virtual screening of drug-like ligands^18–20^. Molecular docking allows for the computational prediction of the three-dimensional structure of protein-ligand complexes (i.e., their binding mode)^21^. Since ligands are flexible molecules that adopt alternative conformations (i.e., different “shapes”), docking tools must consider a ligand’s rotatable bonds (i.e., its internal degrees of freedom, or DoFs), in addition to its position and orientation.

The high-dimensionality of the docking problem prevents an exhaustive exploration of all the DoFs of a ligand at once. Therefore, docking methods implement various heuristics to efficiently explore the ligand’s conformational space and quickly find a low-energy docked conformation of the ligand. For that, binding mode prediction is guided by approximated binding energy calculations (through what is called a scoring function)^21^. Ideally, a docking tool should also be general, in the sense that the accuracy of the predictions should not be impacted by the type of protein receptor or the class of ligands. However, docking methods are known to be much less reliable when applied to larger ligands (e.g., ligands with more than 10 internal DoFs)^22,23^. For instance, peptides are known to be very flexible ligands^24^; binding mode prediction of even small peptides, composed of up to 5 amino acids (which means around 24 internal DoFs), can be particularly challenging for available docking methods^25,26^. This limitation makes the structural prediction of pMHC complexes an impossible task for most docking tools, since a typical MHC-binder is a peptide composed of 8 to 11 amino acids (which translates to more than 30 internal DoFs).

It is worth noting that molecular docking can be used with two distinct objectives: (i) structure-based binding affinity estimation, or (ii) geometry prediction (also referred to as geometry optimization)^14,27^. For instance, recent publications use molecular docking or other structural analyses as part of broader strategies to identify and select MHC-binders (which is known as epitope prediction)^28,29^ or to estimate MHC binding affinity^30–32^. Although they also involve some level of structural prediction, these applications are focused on affinity estimations or approximated ranking of peptides, and are not primarily concerned with providing an accurate 3D model of the pMHC complex. Having a tool for accurate geometry prediction of pMHC complexes would improve the results of structure-based binding affinity predictions, and would enable a number of biomedically-relevant analyses that are not currently available (e.g., pMHC complex stability assessment, structure-based cross-reactivity prediction, etc).

As reviewed in previous publications^14,17^, early attempts at predicting the geometry of pMHC structures usually divided the problem into smaller tasks, as a way to circumvent the limitations of docking tools. For instance, some methods focus on docking the two terminal residues of the peptide, resolving the central portion later^13,14^. Others first approximate the backbone conformation, and then predict the side chains of the peptide^12,33–35^. To make these divisions and approximations, these methods require *ad-hoc* constraints and are usually tailored towards specific MHC allotypes. There also exist methods relying on steps of molecular dynamics (MD)^36,37^. MD can be useful to explore near native conformations of side-chains, but its higher computational cost makes it a less attractive solution for efficient exploration of the entire peptide conformational space^38^. An attempt at a more efficient and yet general solution made use of grid potentials and a biased-probability Monte Carlo method^14^, implemented in the Internal Coordinate Mechanics (ICM) docking tool^39^. This method uses an MHC-specific scoring function trained on available crystal structures via statistical learning. Despite promising results on a small number of known pMHC complexes, the choice of a more specific scoring function and the assumptions on the location of the peptide’s terminal amino acid residues raise questions about the generality of the method towards less prevalent MHC allotypes.

The combination of ICM docking and biased Monte Carlo optimization was also later implemented in pDOCK^16^, and validated on a larger dataset^16^. However, this validation focused on describing the average error of the peptide backbone only, without a broader discussion on the accuracy of side chain predictions. The latest published tool for pMHC structural prediction is DockTope^17^, which uses a protocol based on molecular docking and energy minimization^35^. DockTope was validated on a large dataset of pMHC structures and was the first docking-based method for pMHC prediction to be made available as a webserver. However, it currently provides predictions for only 4 MHC allotypes because it approximates the backbone conformation using allotype-specific patterns from available crystal structures.

An approach similar to that of DockTope was described using the Rosetta FlexPepDock refinement protocol^40^. The authors used available crystal structures of pMHCs as template for peptide backbone conformations, manually positioning the side chains of anchor residues in the expected locations within the binding site. This is justified by the fact that some peptide amino acid residues are known to stabilize the binding by interacting with deeper pockets in the MHC cleft^41^. Then, they conducted a backbone optimization step, followed by side chain prediction. Interestingly, good results were obtained for 5 selected allotypes, even when the template was from a different allotype. However, all reported examples involved complexes that had similar backbone conformations. In addition, the use of backbone templates and assumptions on the position of anchor residues represent important limitations of this method, since they might differ across different groups of MHC allotypes^35,42^. Even for a given MHC allotype, the peptide backbone changes significantly depending on its length. There is also evidence of peptides presenting alternative anchors^42,43^ or unusual binding modes^44^. The FlexPepDock refinement protocol was only applied to 9-mer peptides and a limited number of MHC allotypes. Therefore, it is not yet clear how general this method can be, with respect to these limitations.

Note that most of the aforementioned methods aimed specifically at pMHC predictions. Increasing computational power and growing biomedical interest in peptide ligands and peptide-based inhibitors^45,46^ have fueled the development of new tools for protein-peptide docking in general^47,48^. Some of these tools have been applied to pMHC complexes^49,50^, or validated on datasets including pMHC complexes^51–53^. However, available results are insufficient to make claims on the accuracy and generality of these methods for pMHC structural prediction. For instance, many of these tools were tested using PeptiDB^54^, a dataset of protein-peptide complexes, which, although diverse, includes only one class I MHC-restricted complex. Finally, there are promising tools for *de novo* prediction of protein-peptide complexes^47^, such as Rosetta FlexPepDock *ab-initio*^55^, and HADDOCK peptide docking^56^, which could be applied to pMHC modeling. However, no evaluation of these tools on pMHC complexes has yet been published.

To sum up, to the best of our knowledge, there is yet no general tool for the reliable geometry prediction of pMHC complexes, considering different peptide lengths and MHC allotypes. With the aim of developing a general tool for *de novo* pMHC geometry prediction, in this paper we describe a proof of concept study using an incremental meta-docking approach referred to as DINC (Docking INCrementally)^23^. DINC was previously developed by our group to predict the binding modes of peptidomimetic inhibitors^57^, based on a divide and conquer approach. In contrast to the methods described above, DINC makes no assumption on the location of particular amino acid residues or the shape of the peptide backbone. To evaluate DINC’s applicability and generality in the context of prevalent human MHC allotypes, we performed a re-docking experiment on a diverse dataset of 25 pMHC complexes with available crystal structures. DINC was able to reproduce the binding modes of these complexes with an average error of 1.92 Å. Our results also show the ability of this incremental method to reproduce non-standard binding modes. Finally, we discuss the benefits of having a general tool for pMHC geometry prediction in the growing field of cancer immunotherapy.

## Methods

### Dataset selection

A total of 25 crystal structures of pMHC complexes restricted to human MHC allotypes were selected from the Protein Data Bank (PDB), as listed in the Supplementary Table S1. In humans, MHC receptors are also referred to as Human Leukocyte Antigens (HLAs)^1^. When defining our dataset we prioritized (i) the diversity of peptide sequence and length, (ii) the high prevalence of the HLA allotype in the human population and (iii) the high resolution of the crystal structure. In addition, to analyze an example of T-cell cross-reactivity^10^, we included the recently-determined complexes MAGEA3/HLA-A*01:01 (PDB code 5BRZ) and Titin/HLA-A*01:01 (5BSO). The HLA-B allotypes HLA-B*57:01 and HLA-B*57:03 were also included in our dataset, given their great interest for biomedical purposes (e.g., their role in natural immunity against HIV and HCV)^58,59^ and their different binding modes (as compared to the more prevalent HLA-A*02:01). Finally, an HLA-C complex was included to highlight the generality of the method across the three types of class I HLA.

### Re-docking experiment

This dataset was used for a re-docking experiment, in which we tried to reproduce the binding modes observed in the crystal structures. As a first pre-processing step, all crystal structures were visually inspected and revised as needed^20^. For instance, water molecules were removed since they are not accounted for in our docking method. Also, in cases of duplicated side chains (i.e., conformational heterogeneity), the subset with lower occupancy was removed. Finally, in cases of multiple molecules per asymmetric unit only the first subset was kept (e.g., chains A, B and C). Revised structures were then submitted to a three steps energy minimization with GROMACS v4.6.5^60^, using the steepest-descent and conjugate gradient methods. The GROMOS96 (53a6) force field was used with the SPC water model; a cutoff value of 1.2 nm was used for both van der Waals and Coulomb interactions, with Fast Particle-Mesh Ewald electrostatics (PME). After this procedure, water molecules were removed from the output files and the coordinates of the minimized complexes were saved into PDB format files. These minimized crystal structures will be hereafter referred to as “reference structures”: they are the structures we aim to reproduce in this re-docking experiment. For that, the ligand and receptor in each complex are saved into independent PDB format files, and a docking software is used to reconstruct the original complex. The relevance of re-docking experiments lies in that the conformation and position of an input ligand are systematically randomized by the docking software.

In a re-docking experiment the accuracy of the results is evaluated by assessing the goodness-of-fit between the predicted complexes and the reference structure, usually in terms of Root Mean Square Deviation (RMSD). When computing the RMSD for all atoms of a peptide, between a predicted complex and its reference structure, the two pMHC complexes are first aligned based on the MHC structure. Therefore, the all-atom RMSD captures not only differences in conformation between the two binding modes, but also differences in position of the peptide inside the MHC cleft. While the all-atom RMSD captures changes in both the main chain and the side chains of the peptide, the Cα RMSD (i.e., RMSD for alpha carbons only) captures only changes in the main chain. Another goodness-of-fit measure is the Least Root Mean Square Deviation (LRMSD), computed after aligning the two peptide structures (as opposed to aligning the two MHC structures), either for all atoms or alpha carbons only. The LRMSD is a more precise evaluation of differences between two conformations of a peptide, irrespective of its position in the MHC cleft.

### DINC algorithm

**Figure 2:**
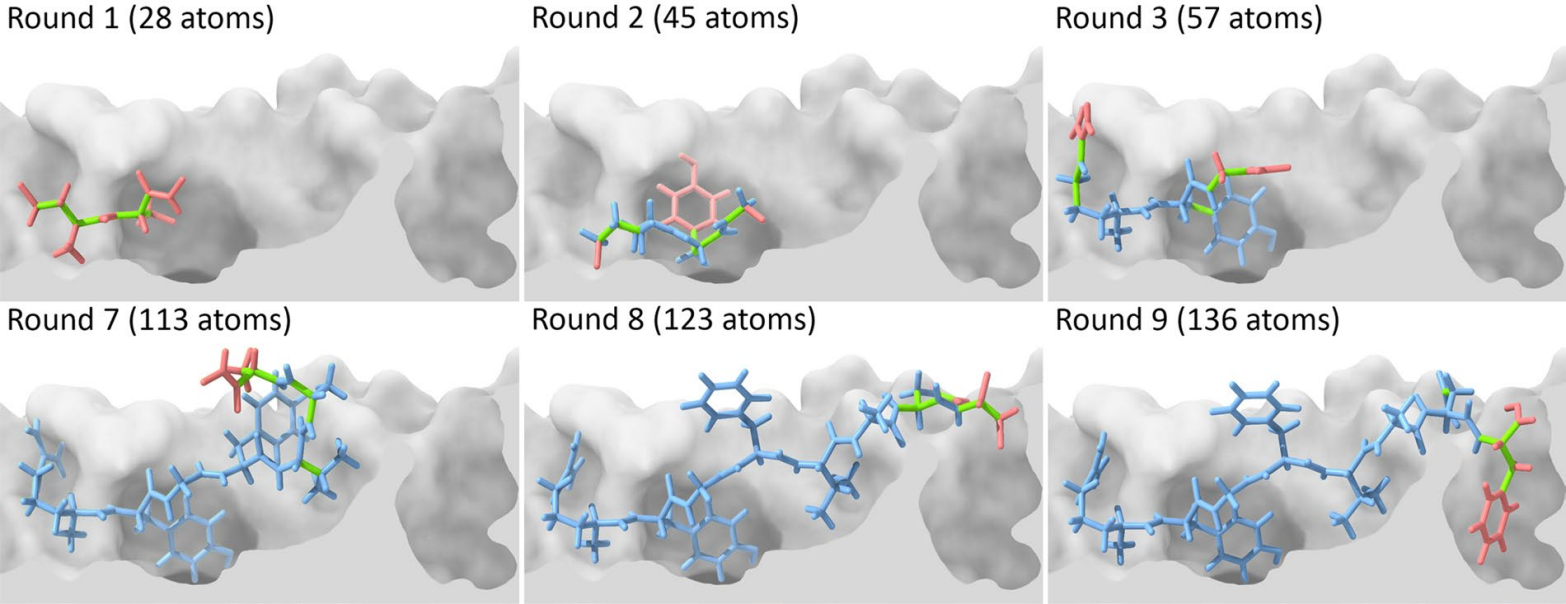
Incremental docking of a peptide. Depiction of some of the docking rounds performed by DINC when re-docking an 8-mer peptide bound to HLA-A*24:02 (PDB code 4F7T). DINC starts by selecting a small fragment of the peptide (top left), with only 6 DoFs (depicted in green), and using it as input for the first round of docking to the MHC binding cleft (cross-section view depicted in gray). The best binding modes are selected across multiple parallel docking runs, and the corresponding peptide fragments are expanded by adding a small number of atoms (depicted in red, top center). These expanded fragments are used as input for the second round of docking (top center), in which a new set of 6 flexible DoFs is considered flexible. These flexible DoFs involve some of the “new” atoms (in red) and some of the atoms that were already present in the previous fragment (in blue). This process continues until the entire ligand has been reconstructed and docked (bottom right). For this particular 8-mer peptide (composed of 136 atoms), DINC has to perform 9 docking rounds; only the first three (top row) and the last three (bottom row) are depicted.

DINC is a parallelized meta-docking method for incremental docking of large ligands, described in detail in previous publications^23,61^. Briefly, instead of docking the entire peptide at once, DINC starts by docking only a small fragment of the peptide (Fig. 2). The best conformation for this “initial fragment” is selected using a scoring function, and expanded through the addition of another subset of atoms from the original peptide. This new expanded fragment is then docked, and this process is incrementally repeated until the entire peptide is reconstructed and docked. Note that the word “fragment” is used here with a different meaning than that of fragment-based drug discovery (FBDD). FBDD uses libraries of fragments, which are very small molecules with no more than two functional groups^62^, to create new drugs or drug-like ligands. DINC was inspired by these methods, but does not use any library of small molecules, and does not dock independent fragments that are later connected. In the context of DINC, the fragments are overlapping sections of the input ligand, docked sequentially to grow the ligand incrementally^61^.

DINC currently uses the standard docking software AutoDock 4^20,63^; a free online version of DINC is available as a webserver (http://dinc.kavrakilab.org/)^61^. While DINC manages the fragment selection and expansion, as well as the parallelization of the search, AutoDock 4 performs the sampling and scoring of individual fragments. More specifically, AutoDock 4 uses a Lamarckian genetic algorithm^63^. Genetic algorithms are a type of evolutionary technique that is commonly used for the stochastic sampling of ligand conformations in molecular docking^64^. For scoring, AutoDock 4 uses a semi-empirical free energy force field, including terms for dispersion/repulsion, hydrogen bonding, electrostatics and desolvation^20,63^. In this paper we used a custom version of DINC, to explore different parameters (e.g., number of DoFs at each round) and heuristics (e.g., fragment expansion method).

### Experimental setup

**Figure 3:**
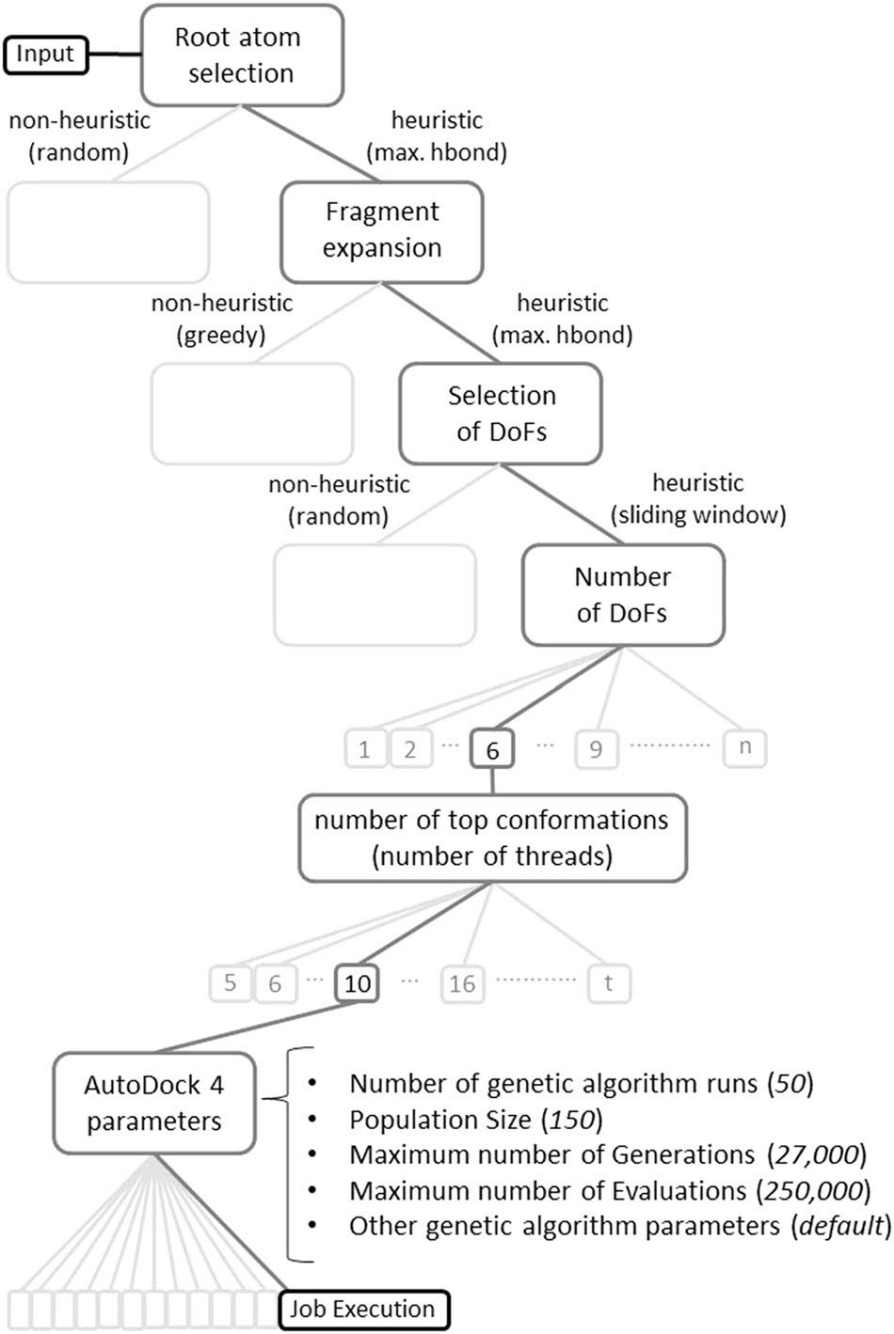
Default protocol for a DINC job. As highlighted in this decision tree, the default DINC protocol selects the root atom using a heuristic maximizing the potential for hydrogen bonds in the initial fragment (max. hbond), by counting the number of available donors and acceptors; expands the fragment at each round using the same heuristic; selects potential rotatable bonds for sampling based on a sliding window approach and activates only 6 DoFs at each round (*see* Fig. 2); selects the top 10 conformations for expansion (ranked by binding energies); and uses default values for AutoDock 4 parameters (indicated within parenthesis). Alternative protocols can be defined for a DINC job, by making different choices in this decision tree.

DINC is a customizable approach, allowing for the use of different combinations of parameters and heuristics. In this context, one set of parameters chosen for a particular job (i.e., one execution of DINC) is referred to as a DINC protocol. From previous experience, we define a default protocol for DINC (Fig. 3). This protocol represents a reasonable selection of parameter values for a standard job, but it is not expected to provide the best results in all situations. Therefore, in our re-docking experiment, four alternative protocols were defined, exploring other combinations of parameter values. The specific parameter values in these five protocols (including the default protocol) are presented in Supplementary Table S2. It is important to highlight that we do not exhaustively evaluate all possible protocols. For example, DINC protocols also comprise the specific parameters of the underlying docking software, in this case AutoDock 4 (Fig. 3). In our experiment, default values were used for AutoDock 4 parameters (e.g., ga_run = 50, pop_size = 150, num_evals = 250000, num_gen = 27000, elitism = 1, mutation_rate = 0.02, crossover_rate = 0.8, etc).

The five protocols are used in the following way: First, a total of 20 DINC jobs is executed for each of the 25 complexes in the dataset (Supplementary Table S1), using the default protocol (Fig. 3). The output conformation with the lowest binding energy for each complex is compared to the corresponding reference structure. If the all-atom RMSD between them is lower than 2 Å, this is considered a good reproduction of this complex. If not, a new batch of 20 DINC jobs is executed using the next alternative protocol. This process is repeated until a good reproduction is obtained, or the five protocols have been used (which corresponds to 100 jobs).

Finally, it is important to remember that the docking search is not biased by the reference structure. Additionally, each new DINC job is completely independent from all previous jobs. All 20 jobs of a given batch are executed in parallel. Our cluster contains 80 dual processor HP SL230s computing nodes, each one equipped with two Intel E5-2650v2 Ivy Bridge EP processors (for a total of 16 cores per node). The typical running time for a DINC job on our cluster is 30 minutes (for a CPU time of about 8 hours).

### Visualization

Cartoon representations of the MHCs, cross-section views of the binding clefts, and side view images of the peptides were obtained with the UCSF Chimera^65^ and UCSF ChimeraX packages. These packages are developed by the Resource for Biocomputing, Visualization, and Informatics at the University of California, San Francisco (supported by NIGMS P41-GM103311). Molecular surfaces of pMHC complexes were computed with GRASP2^66^, which was also used to obtain top view images of these complexes. Electrostatic potentials were computed with Delphi for a range of −5 kT/e (red) to +5 kT/e (blue), using the GRASP2 interface.

### Data availability

The datasets generated and analyzed during the current study are available from the corresponding author on reasonable request.

## Results and Discussion

### Re-docking of a diverse dataset of pMHC complexes

**Figure 4:**
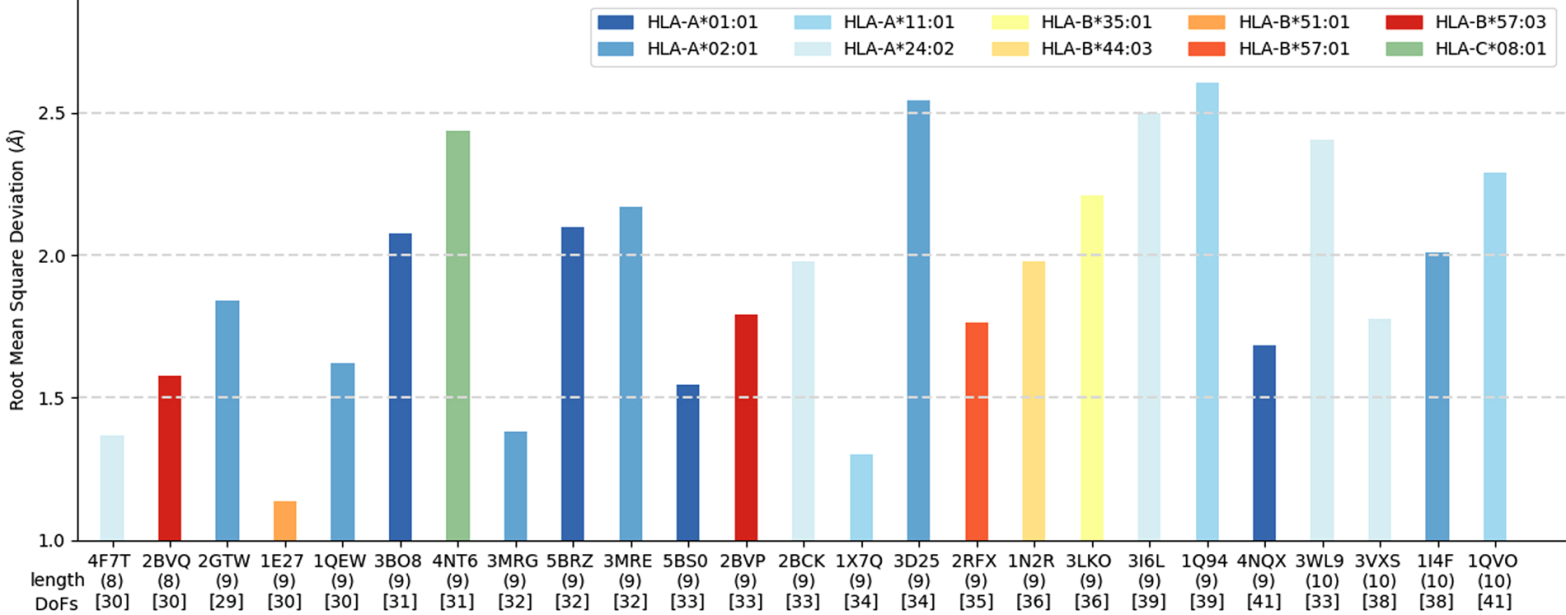
Re-docking of 25 peptides bound to prevalent human MHC allotypes. Each bar indicates the all-atom Root Mean Square Deviation (RMSD) between the reference structure and the best binding mode predicted by DINC (*see* Methods). Results are sorted by increasing peptide length, then number of DOFs, then RMSD. The peptide length and number of DoFs are listed between parenthesis and between brackets, respectively. Complexes are identified by their PDB codes.

We performed a re-docking experiment with a diverse dataset of pMHC complexes (Supplementary Table S1). Our dataset includes 10 of the most prevalent human MHC allotypes, bound to peptides with different lengths (8 to 10 amino acids) and a varying number of DoFs (29 to 41). The goodness-of-fit between predicted binding modes and the corresponding reference structures (i.e., the minimized crystal structures) is estimated in terms of RMSD. A “near native” reproduction of an experimentally-observed binding mode usually corresponds to an all-atom RMSD lower than 2.0–2.5 Å^17,47^. The results of our re-docking experiment show good reproductions of the 25 pMHC complexes (Fig. 4), with an average all-atom RMSD of only 1.92 Å (±0.41 Å). The all-atom RMSD is less than 2.2 Å in 68% of the cases, and less than 2.5 Å in 98%; the highest all-atom RMSD is 2.61 Å. Note that most previous work in the field was reported using backbone (or Cα) RMSD only^13,14,16,36,50^. This means capturing the overall “shape” of reproduced peptides, but not necessarily the precise position of their side-chains. On the other hand, related work reporting all-atom RMSD was usually performed in less diverse datasets (e.g., only selected MHC allotypes or peptide lengths)^17,34,37,40^. We report both Cα RMSD and all-atom RMSD for our re-docking experiment, which was conducted on a structurally diverse dataset of pMHC complexes (Supplementary Table S1).

As seen from Fig. 4, we do not observe any correlation between the all-atom RMSD and the number of DoFs (*R* = 0.39). For instance, the result for complex 4NT6 (9-mer, 31 DoFs) is worse than for complex 3VXS (10-mer, 38 DoFs). When considering all the variables listed in Supplementary Table S1, the strongest correlation (*R* = 0.5) is observed between peptide length and Cα LRMSD. By computing the Cα LRMSD we capture the accuracy of the backbone prediction. This is very meaningful information because a small error in the backbone has a bigger impact on the binding mode than a similar error in a side chain.

DINC makes no initial assumption on the backbone conformation, and has no constraint related to templates or expert-knowledge on the expected conformation (Fig. 2). In spite of that, our average Cα LRMSD is only 0.99 Å (±0.36 Å). As further discussed in the next section, a good reproduction of the backbone is obtained even when considering peptides with different lengths, or “non-standard” binding modes. Besides, similar levels of accuracy are obtained for very different MHC allotypes, highlighting the potential of DINC as a general method for pMHC structural prediction.

### Accurate prediction of diverse binding modes

**Figure 5:**
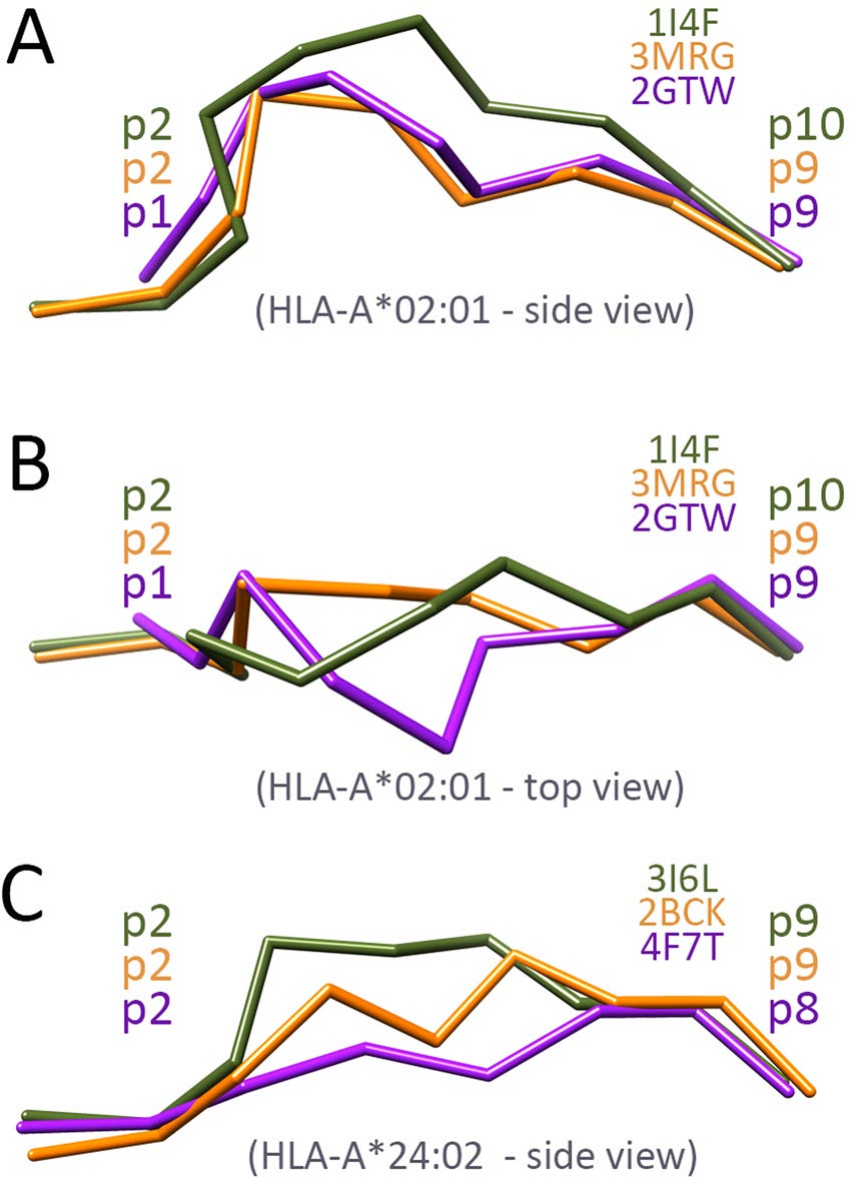
Alternative peptide backbone patterns. Schematic backbone representations (*chain trace*) of 6 different peptides, experimentally observed bound to either HLA-A*02:01 (A, B) or HLA-A*24:02 (C). (**A**) Side views of a typical HLA-A*02:01-restricted virus-derived 9-mer peptide (PDB code 3MRG, depicted in orange), a tumor-derived 9-mer peptide with an alternative binding mode (2GTW, in purple), and a tumor-derived 10-mer peptide (1I4F, in green). (**B**) Top views of the same HLA-A*02:01-restricted peptides. (**C**) Side views of a typical HLA-A*24:02-restricted virus-derived 9-mer peptide (2BCK, orange), a shorter 8-mer peptide (4F7T, purple), and a virus-derived 9-mer with an alternative binding mode (3I6L, green).

**Figure 6:**
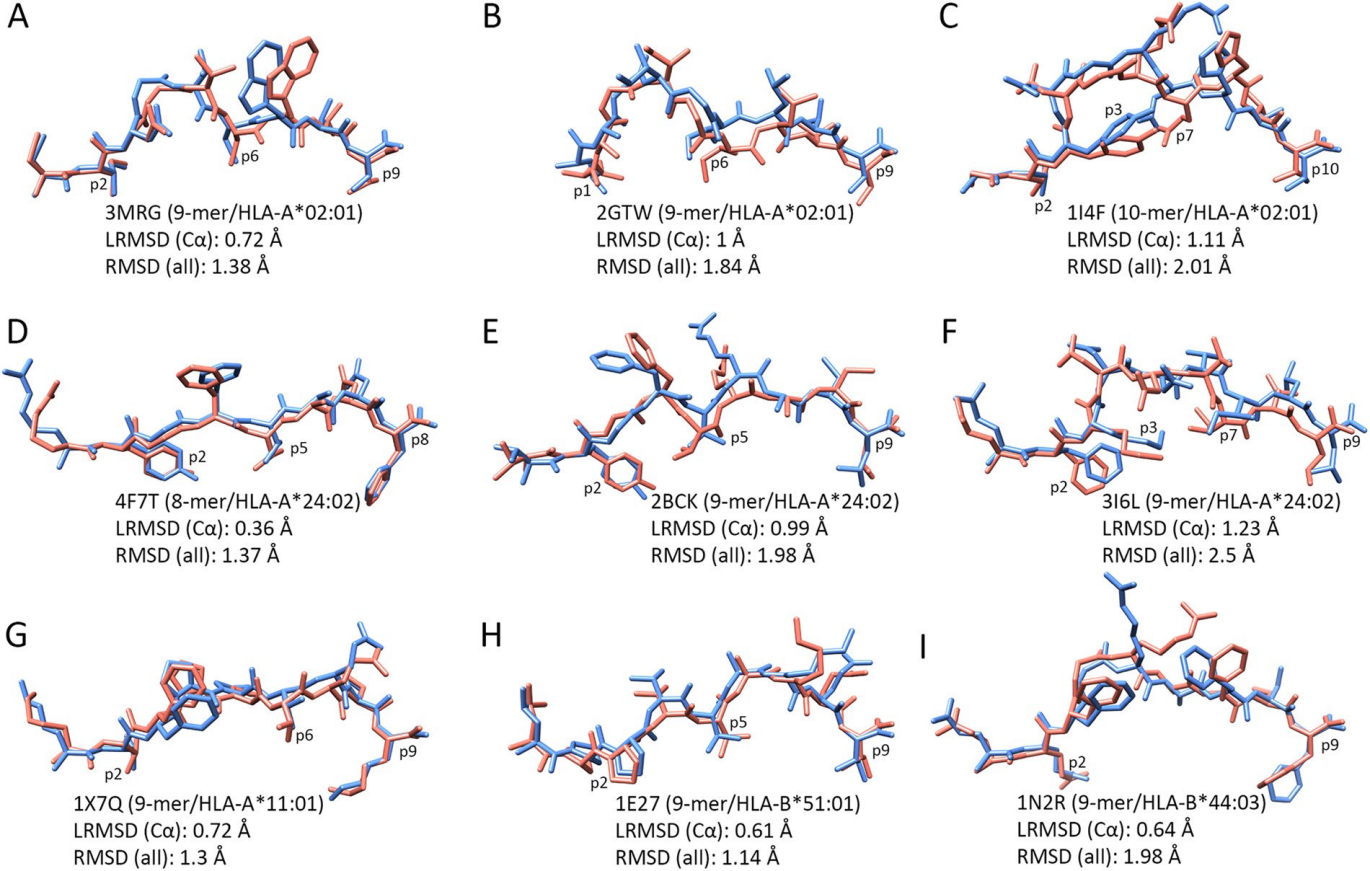
Reproduction of very different binding modes. In blue, side view of nine different peptides bound to five different human MHC allotypes, as observed in the corresponding reference structures (identified by their respective PDB codes). In pink, side view of the best binding modes obtained by DINC when performing a re-docking experiment with each complex. The MHC structure is not depicted, but the HLA allotype is indicated for each complex. Note that alternative peptide residues can be involved as primary anchors (p1/p2, p8/p9/p10) or secondary anchors (p3, p5, p6, p7), depending on the peptide length or MHC allotype. LRMSD (Cα), Least Root Mean Square Deviation for the alpha carbons of the peptide; RMSD (all), Root Mean Square Deviation for all atoms of the peptide. Additional information can be found in Supplementary Table S1.

The allotype HLA-A*02:01 is one of the most extensively studied HLA variants^17,41^. It is the second most prevalent allotype in humans^67,68^ and arguably the HLA variant for which the most detailed and comprehensive data is available: more than 42,000 binding assays deposited in the Immune Epitope Database^69^. As reported in previous studies, HLA-A*02:01 binds mostly 9-mers, but also larger peptides. It was also reported that the HLA-A*02:01 binding cleft is fairly constrained, with little conformational variation across available crystal structures. It also presents a clear pattern of preferred anchor residues^41^ at both peptide termini: usually positions 2 (p2) and 9 (p9) of the 9-mer ligands (Fig. 5). Comparing available crystal structures, a shared conformational pattern was observed for the backbone of 9-mer peptides bound to HLA-A*02:01^35^. For example, this typical backbone pattern is observed in the crystal structure 3MRG, involving a virus-derived peptide (Fig. 5A). Some cancer-related peptides, however, are known to present unusual binding modes^17^. For instance, in the modified melanoma-associated antigen MART1-A27L the amino-terminal anchor to the HLA-A*02:01 binding cleft is p1 instead of p2. This alternative anchoring pattern creates a sideways deviation of the backbone in the middle of the peptide^17^, resulting in an unusual binding mode (Fig. 5B, 2GTW). In addition, larger peptides are known to present bulging conformations of the backbone, to accommodate a longer chain using the same anchoring pockets (Fig. 5A, 1I4F). DINC was able to reproduce each one of these 3 alternative backbone conformations, with sub-angstrom accuracy (Fig. 6).

The most prevalent HLA allotype is HLA-A*24:02^67,68^, which is known to bind 8-mers, 9-mers and 10-mers. As expected, the conformation of 8-mers is more linear, with almost no bulged region between the two peptide termini (Fig. 5C, 4F7T). Currently, there are only two crystal structures of 9-mers bound to HLA-A*24:02, and they present different binding modes (Fig. 5C). According to the researchers who described these structures, the virus-derived peptide resolved in 3I6L shows an unusual binding mode, with a much more exposed p4 as compared to a regular self-derived peptide (2BCK). Further analysis of other crystal structures shows that the backbone conformation seen in 3I6L is very similar to that of 10-mer peptides bound to the same HLA (data not shown). In our re-docking experiment, DINC was able to reproduce all these binding modes (Fig. 6), as well as other 10-mers bound to HLA-A*24:02 (Supplementary Table S1). In the case of 3I6L, the authors claim that the observed binding mode is stabilized by an internal hydrogen bond established by p3, whose side chain is pointing towards the center of the binding cleft. The binding mode predicted by DINC for this complex does not feature this specific hydrogen bond (as determined by UCSF Chimera), but a similar orientation of p3 is observed (Fig. 6F).

The peptide’s binding mode is greatly influenced by the shape and properties of the HLA cleft; a given peptide might bind differently to different HLA allotypes (e.g., using different anchor residues or having different side chains exposed for TCR interaction)^35,70^. These structural differences are key for recognition by T-cells, and contribute to the diversity of cellular responses observed among individuals with different subsets of HLAs^1^. Although our dataset includes peptides bound to four different HLA-A allotypes, five HLA-B allotypes and one HLA-C allotype (Supplementary Table S1), we can reproduce the conformational differences imposed by these different binding clefts (Fig. 6).

The results in this paper show that it is possible to develop a general pMHC geometry prediction method. In addition to the good reproduction of peptides’ backbone, the average all-atoms LRMSD of 1.73 Å (±0.33 Å) demonstrates high accuracy reproduction of peptides’ side chains (Supplementary Table S1). Therefore, not only the buried side chains (i.e., those facing the MHC cleft) were correctly predicted, but the overall geometry of the ligand was closely reproduced (including side-chains of the bulging portion of the peptide, which are more exposed for TCR interaction). Obtaining a good approximation of the pMHC complex geometry is essential for the use of predicted models as input for other structure-based analyses.

### Significance for T-cell-based immunotherapy

Thanks to the rapid technical developments of the last decade and our growing understanding of the mechanisms involved in cellular immunity, T-cell-based immunotherapy has emerged as one of the most promising approaches for cancer treatment^7,71,72^. Significant anti-tumor activity has been reported in a number of clinical trials, involving different cancer types^73^. Two melanoma-associated antigens, MAGEA3 and MART1, stand out among the leading tumor-derived peptides targeted by these immunotherapies (Supplementary Table S1).

The peptide-antigens derived from MAGEA3 and MART1 can be expressed by multiple tumor types, but are not expressed by most normal tissues, therefore allowing for the development of antigen-specific T-cell-based therapeutics^74–76^. Unfortunately, unexpected off-target toxicities against healthy tissues have been reported^74–76^, raising serious safety concerns. For instance, lethal cardiac toxicity was observed in two patients undergoing treatment with T-cells specific to the MAGEA3 antigen^74,75^. Later investigation showed that the therapeutic T-cells used in these patients were also recognizing an unrelated Titin-derived peptide, displayed by HLA-A*01:01 molecules in healthy cardiac cells^74,75^.

**Figure 7:**
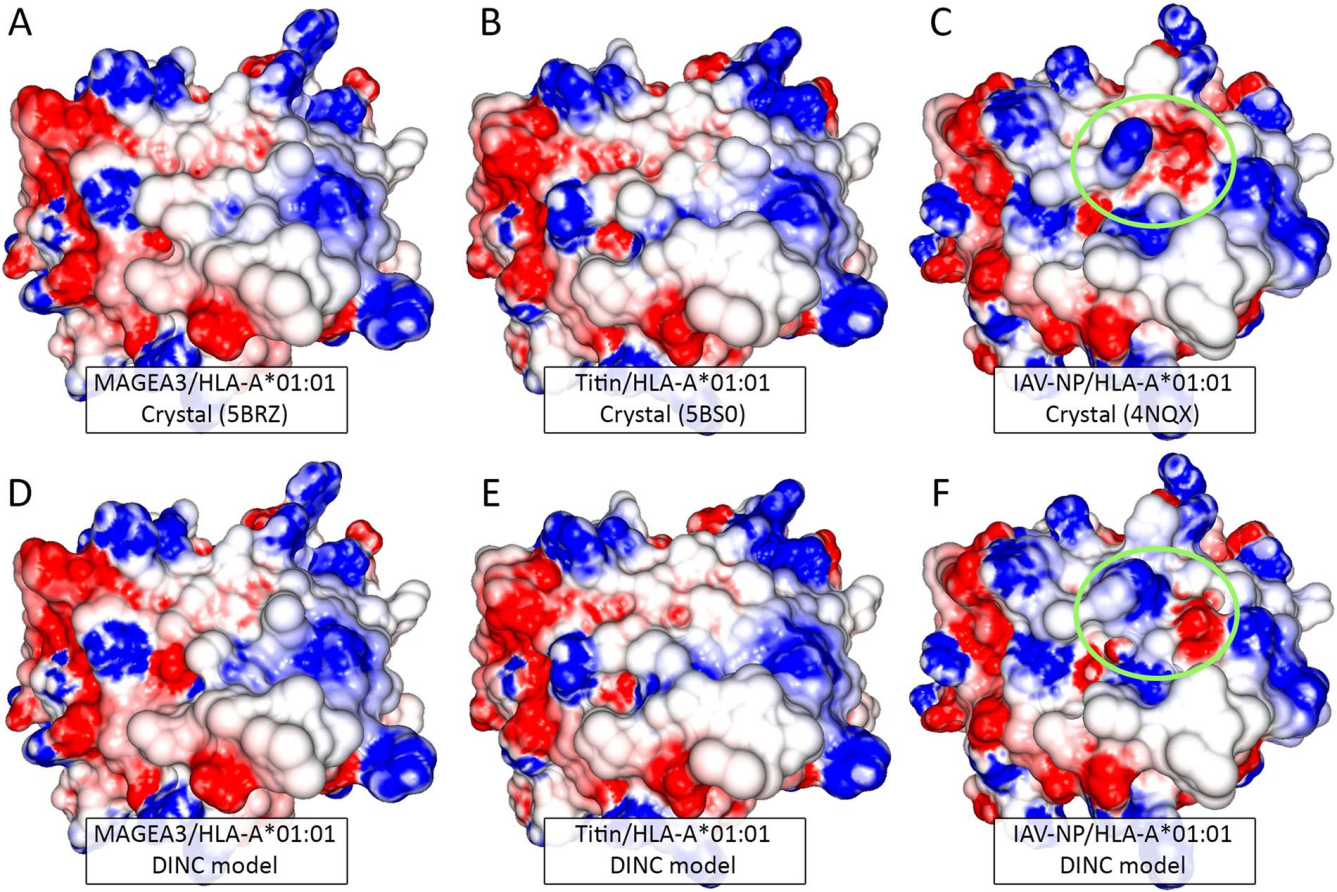
TCR-interacting surface of cross-reactive pMHC complexes. Cross-reactivity was reported between the melanoma-associated antigen MAGEA3 (EVDPIGHLY) and a Titin-derived self peptide (ESDPIVAQY). Crystal structures (depicted in the top row and referenced by their PDB code) show the structural similarity of these peptides when bound to HLA-A*01:01 (**A**,**B**). As a comparison, a virus-derived peptide bound to the same MHC presents differences in both topography and charge distribution (**C**, most significant differences indicated by a green circle). The DINC models (depicted in the bottom row) reproduce the structural similarities of the two cross-reactive complexes (**D**,**E**); and the model for the virus-derived peptide reproduces its differences (**F** green circle). IAV, Influenza A Virus.

Although we usually refer to the peptides as being the targets recognized by the cytotoxic T-cells, TCRs actually recognize the combined surface of the peptide and MHC receptor, also referred to as the “TCR-interacting surface” of the pMHC complex (Fig. 1). Each TCR is thought to be specific for a given pMHC complex, but structural similarity between unrelated complexes can be responsible for off-target activation of T-cells^8,77^; also known as T-cell cross-reactivity^6^. Using x-ray crystallography, Raman and colleagues^10^ have confirmed the structural similarity between the pMHC complexes involved in the MAGEA3-Titin cross-reactivity (Fig. 7A,B). Both of these pMHC complexes were included in our dataset (5BRZ and 5BS0). DINC was able to correctly predict the geometry of both peptides, bound to HLA-A*01:01, and reproduce the structural similarity of the resulting TCR-interacting surfaces (Fig. 7D,E).

The clinically relevant example described above highlights the significance of pMHC structural prediction in the context of T-cell-based immunotherapy. In this case, the two peptides have a sequence identity of 55%, which is already challenging for sequence-based cross-reactivity prediction. However, T-cell cross-reactivity can be triggered even by peptides with no sequence identity and low biochemical similarity^78^, and might be driven by specific structural similarities in hot-spots over the TCR-interacting surface^79^. In this context, structure-based methods for cross-reactivity prediction have been proposed, either clustering pMHCs of interest based on structural similarity^78,80^, or integrating structural information and protein expression levels into sequence-based proteomic searches^81,82^. This field will spawn significant developments in the coming years, particularly considering the importance of cross-reactivity prediction for T-cell-based immunotherapy^11^. Moreover, considering the costs and practical limitations of experimental methods for protein structural analysis, fast and reliable computational methods for geometry prediction of pMHC complexes should play an important role in this process.

## Current challenges and future work

The high-dimensionality of the search space is a challenge inherent to molecular docking. Algorithmic solutions to address this challenge are usually non-deterministic, introducing variability, which affects reproducibility. For instance, a single run of AutoDock 4 starts with a random conformation of the ligand, which is then randomly modified by the Lamarckian genetic algorithm to create new conformations^20,63^. Therefore, the chances of obtaining different results in independent runs of AutoDock 4 increase with ligand size. A similar variability is observed across independent DINC jobs. However, this is not a problem here, as our goal was only to determine if DINC could predict different binding modes of pMHC complexes within a reasonable time. By providing a proof of concept that this goal is indeed attainable, we can now open new avenues for developing even better algorithms inspired by the meta-docking incremental approach. In fact, since only a few protocols were used in the context of this study, there is great potential for further improvement. As future work, we will perform a thorough evaluation of the parameters and heuristics in DINC in order to improve its efficiency and achieve fast, accurate and reproducible geometry prediction of pMHC complexes.

The general structural prediction of pMHC complexes requires addressing a combination of challenges, including peptide-docking, receptor flexibility and accurate scoring. Here, we focused on the peptide-docking problem, and showed how a simple incremental approach allows predicting binding modes of peptides with different lengths and bound to different MHCs. For that, we limited our analysis to a re-docking experiment involving a diverse dataset of human pMHCs. Receptor flexibility is another important challenge^27^ and should be taken into consideration when predicting pMHC complexes^83^. In fact, MHC flexibility can affect peptide loading, and peptide binding can induce local changes in the MHC receptor^84,85^. However, the folding of MHC receptors is highly-conserved. Therefore, a docking protocol accounting for receptor flexibility could be combined with homology modeling to predict the binding modes of peptides to MHC allotypes for which no structural information is available^14,16^. However, the high-dimensionality of the resulting search space requires even better algorithms, which may involve dimensionality reduction approaches^86^.

Recent reviews indicate that other docking software can outperform AutoDock 4 in scoring predicted binding modes^26^. In fact, scoring is one of the bottlenecks when trying to achieve greater accuracy in docking-based methods^26^. In terms of sampling, our method could certainly provide results with sub-angstrom accuracy, but this would require a scoring function capable of discriminating between conformations with sub-angstrom differences. Therefore, our method could benefit from using consensus scoring^87^, peptide-specific scoring^88^ or HLA-specific scoring^89^. Being a meta-docking application, DINC can integrate alternative sampling strategies or scoring methods. We plan to further investigate these issues in a future study, performing cross-docking and benchmarking on a much larger pMHC dataset.

Finally, a version of DINC with improved scoring could also provide a more general tool for epitope prediction and virtual screening of MHC binders. Gold standard tools for these tasks usually rely on machine learning methods trained on available datasets of previously tested peptide sequences^90^, which are limited or inexistent for less prevalent MHC allotypes^91,92^. DINC do not require *ad-hoc* knowledge on the typical binders for a given MHC allotype, or its preferred primary anchors. Therefore, once the aforementioned challenges are addressed, DINC could potentially complement sequence-based methods in epitope prediction projects, by providing structure-based ranking of peptide-ligands for any MHC of interest.

## Conclusion

In this paper, we demonstrate that an incremental meta-docking approach can predict the binding modes of large peptide ligands bound to MHC receptors. Standard docking software can provide general solutions (i.e., solutions that are not restricted to a particular protein receptor), but cannot handle large ligands. On the other hand, methods focused on pMHC structural prediction lack generality because they often use expert-knowledge or frequent patterns as constraints. We argue that the use of incremental docking offers a new strategy to overcome these limitations. Our work shows that incremental docking allows handling different MHC allotypes, predicting unusual binding modes, and obtaining accurate structural prediction for peptides with up to 41 rotatable bonds. In addition, being a meta-docking approach, our method avoids the need for new docking software. We postulate that a similar incremental process could be implemented using different docking software, achieving similar or even better results. As a proof of concept, our study represents a landmark in the advancement of methods for geometry prediction of pMHC complexes. Future developments of these methods are expected to have a positive impact in many fields related to human health, including vaccine development and tissue transplantation. In particular, fast and accurate prediction of patient-specific pMHC complexes will be key for the development of safe and effective T-cell-based immunotherapies against cancer.

## Electronic supplementary material

Supplementary Information
