# Supplementary material for: General Prediction of Peptide-MHC Binding Modes Using Incremental Docking: A Proof of Concept
Source: Sci Rep. 2018 Mar 12;8:4327. doi: 10.1038/s41598-018-22173-4 (PMC5847594; doi:10.1038/s41598-018-22173-4)
Supplement: Supplementary file 1 — Supplementary Information [file 41598_2018_22173_MOESM1_ESM.pdf]

General Prediction of Peptide-MHC Binding Modes Using Incremental Docking: A Proof of Concept

Dinler A. Antunes[1], **Didier Devaurs**[1], **Mark Moll**[1], **Gregory Lizée**[2], **and Lydia E. Kavraki**[1,*]

[1]Department of Computer Science, Rice University, Houston, TX 77005, USA
[2]Department of Melanoma Medical Oncology - Research, The University of Texas M.D. Anderson Cancer Center, Houston, TX 77054, USA
[*]kavraki@rice.edu

Supplementary Information File

Supplementary Table S 1. Dataset of pMHC complexes and corresponding re-docking results.

MHC Allotype	Peptide	Organism	Antigen	IEDB	PDB	Length	DoFs	Resolution	LRMSD (Cα)	LRMSD (all)	RMSD (Cα)	RMSD (all)
HLA-A*24:02	RYGFVANF	IAV	PB1 (498-505)	124692	4F7T	8	30	1.70	0.36	1.28	0.60	1.37
HLA-B*57:03	KAFSPEVI	HIV-1	Gag (30-37)	187159	2BVQ	8	30	2.00	0.54	1.49	0.76	1.58
HLA-A*02:01	LAGIGILTV	H. sapiens	MART1-A27L (27-35)	99827	2GTW	9	29	1.55	1.00	1.55	1.36	1.84
HLA-B*51:01	LPPVVAKEI	HIV-1	Gag-Pol (747-755)	180337	1E27	9	30	2.20	0.61	1.10	0.73	1.14
HLA-A*02:01	FLWGPRALV	H. sapiens	MAGEA3 (271-279)	16970	1QEW	9	30	2.20	0.75	1.57	0.98	1.62
HLA-A*01:01	EADPTGHSY	H. sapiens	MAGEA1 (161-169)	11010	3BO8	9	31	1.80	1.33	1.92	1.57	2.08
HLA-C*08:01	GILGFVFTL	IAV	M1 (29-37)	20354	4NT6	9	31	1.84	1.35	2.15	2.23	2.44
HLA-A*02:01	CINGVCWTV	HCV	NS3 (1073-1081)	6435	3MRG	9	32	1.30	0.72	1.18	0.90	1.38
HLA-A*01:01	EVDPIGHLY	H. sapiens	MAGEA3 (168-176)	14672	5BRZ	9	32	2.62	1.40	1.87	1.94	2.10
HLA-A*02:01	GLCTLVAML	EBV	BMLF1 (259-267)	20788	3MRE	9	32	1.10	1.37	2.05	1.61	2.17
HLA-A*01:01	ESDPIVAQY	H. sapiens	Titin (24337-24345)	509235	5BS0	9	33	2.40	0.46	1.37	0.71	1.55
HLA-B*57:03	ISPRTLDAW	HIV-1	Gag (147-158)	-	2BVP	9	33	1.35	0.92	1.68	1.05	1.79
HLA-A*24:02	VYGFVRACL	H. sapiens	TERT (461-469)	99851	2BCK	9	33	2.80	0.99	1.88	1.02	1.98
HLA-A*11:01	KTFPPTEPK	SARS-CoV	Nucleoprotein (362-370)	33667	1X7Q	9	34	1.45	0.72	1.23	0.83	1.30
HLA-A*02:01	VLHDDLLEA	H. sapiens	HMHA1 (137-145)	136896	3D25	9	34	1.30	1.07	2.08	1.73	2.54
HLA-B*57:01	LSSPVTKSF	H. sapiens	IGKC (93-101)	98894	2RFX	9	35	2.50	0.93	1.60	1.08	1.76
HLA-B*44:03	EEFGRAFSF	H. sapiens	HLA-DPA1 (46-54)	95009	1N2R	9	36	1.70	0.64	1.94	0.70	1.98
HLA-B*35:01	LPFDRTTIM	IAV	Nucleoprotein (418-426)	38468	3LKO	9	36	1.80	1.28	1.88	1.66	2.21
HLA-A*24:02	QFKDNVILL	SARS-CoV	Nucleoprotein (346-354)	50779	3I6L	9	39	2.40	1.23	2.12	1.56	2.50
HLA-A*11:01	AIFQSSMTK	HIV-1	Gag-Pol (263-271)	1913	1Q94	9	39	2.40	1.37	2.14	2.03	2.61
HLA-A*01:01	CTELKLNDY	IAV	Nucleoprotein (44-52)	181983	4NQX	9	41	2.30	0.79	1.54	1.20	1.68
HLA-A*24:02	NYTPGPGIRF	HIV-1	Nef (129-138)	230028	3WL9	10	33	1.66	1.96	2.23	2.43	2.41
HLA-A*24:02	RYPLTLGWCF	HIV-1	Nef (133-142)	193071	3VXS	10	38	1.80	0.98	1.73	1.31	1.78
HLA-A*02:01	GVYDGREHTV	H. sapiens	MAGEA4 (230-239)	95091	1I4F	10	38	1.40	1.11	1.91	1.26	2.01
HLA-A*11:01	QVPLRPMTYK	HIV-1	Nef (73-82)	52760	1QVO	10	41	2.22	0.99	1.81	1.37	2.29
							Mean	1.91	0.99	1.73	1.30	1.92
							SD	0.470	0.365	0.329	0.506	0.413

Peptide-MHC complexes are sorted (from top to bottom) by increasing peptide length, then number of DOFs, then RMSD (all). Resolution and deviation values are provided in Å. RMSD, Root Mean Square Deviation; LRMSD, Least Root Mean Square Deviation; Cα, alpha carbons; "all", all peptide atoms; DoFs, Degrees of Freedom corresponding to all rotatable bonds, except amide bonds and guanidinium bonds; SD, Standard Deviation. The protein source for each peptide can be found in the Antigen column, with the peptide position provided between parenthesis. IAV, Influenza A Virus; HIV-1, Human Immunodeficiency Virus type 1; EBV, Epstein-Barr Virus; HCV, Hepatitis C Virus; SARS-CoV, Severe Acute Respiratory Syndrome-associated Coronavirus.

Supplementary Table S 2. **Five alternative DINC protocols**.

	Protocol 1	Protocol 2	Protocol 3	Protocol 4	Protocol 5
Root atom selection	max. hbond	random	max. hbond	max. hbond	max. hbond
Fragment expansion	max. hbond	greedy	greedy	max. hbond	greedy
Selection of DoFs	sliding window	sliding window	sliding window	random	random
Number of DoFs	6	6	6	9	12
Number of top conformations	10	16	16	16	16
AutoDock 4 parameters	default	default	default	default	default

Protocol parameters are listed in the same order presented in Fig. 3. max. hbond, heuristic for maximizing the number of hydrogen bonds in the fragment; greedy, breadth-first algorithm for expansion; sliding window, heuristic for DoF selection in which are sampled only bonds connecting atoms added in the current (j) or previous (j-1) rounds of a DINC job (*see* Fig. 2). Default values for AutoDock 4 included: Genetic Algorithm runs = 50, Population Size = 150, Maximum number of Generations = 27000, Maximum number of Evaluations = 250000, etc.
